# Sedentary Patterns Are Associated with Bone Mineral Density and Physical Function in Older Adults: Cross-Sectional and Prospective Data

**DOI:** 10.3390/ijerph17218198

**Published:** 2020-11-06

**Authors:** Luís Alberto Gobbo, Pedro B. Júdice, Megan Hetherington-Rauth, Luís B. Sardinha, Vanessa Ribeiro Dos Santos

**Affiliations:** 1 Skeletal Muscle Assessment Laboratory, Department of Physical Education, School of Technology and Sciences, São Paulo State University, São Paulo 19060-900, Brazil; luis.gobbo@unesp.br; 2 Post-Graduation Program in Movement Sciences, School of Technology and Sciences, São Paulo State University, São Paulo 19060-900, Brazil; 3 Exercise and Health Laboratory, CIPER, Faculdade de Motricidade Humana, Universidade de Lisboa, 1649-004 Cruz-Quebrada, Portugal; judicepd@gmail.com (P.B.J.); mchr@email.arizona.edu (M.H.-R.); lbsardinha55@gmail.com (L.B.S.); 4 Centro de Investigação em Desporto, Educação Física e Exercício e Saúde, CIDEFES, Universidade Lusófona, 1749-024 Lisbon, Portugal

**Keywords:** acceleration, bone health, exercise, physical health, sedentary living

## Abstract

Aging causes some unfavorable morphological and functional changes, such as the decline in bone mineral density (BMD) and physical function. Moderate-to-vigorous physical activity (MVPA) and sedentary time seem to be related with these alterations, but the impact of distinct patterns remains unclear. The aim of this study was to cross-sectionally and prospectively assess the association between objectively measured MVPA and sedentary patterns (bouts and breaks) with BMD and physical function in older adults. The study considered 151 Brazilians (aged ≥ 60 years), out of which 68 participants completed 2-year follow-up measurements. MVPA and sedentary patterns were measured by means of accelerometry, BMD—(total proximal femur and lumbar spine (L1-L4)) by means of dual-energy X-ray absorptiometry (DXA), and physical function—by means of physical tests. In older women, sedentary bouts >60 min were inversely associated with handgrip strength (β = −2.03, 95% CI: from −3.43 to −0.63). The prospective analyses showed that changes in sedentary bouts (20 to 30 min and >60 min) were inversely associated with changes in the lumbar spine’s BMD (β = −0.01, 95% CI: from −0.01 to −0.00 and β = −0.03, 95% CI: from −0.06 to −0.01) and the lumbar spine’s T-score (β = −0.06, 95% CI: from −0.10 to −0.01 and β = −0.27, 95% CI: from −0.49 to −0.04), respectively. In older women, sedentary patterns are cross-sectionally associated with handgrip strength and prospectively associated with BMD independent of MVPA.

## 1. Introduction

The natural process of aging causes some unfavorable morphological and functional changes, such as the decline in bone mineral density (BMD) [1] and physical function [2], which increase the risk of developing disorders such as osteoporosis and functional disability.

Behavior patterns such as moderate-to-vigorous physical activity (MVPA) [3] and sedentary time seem to be related to these alterations [4,5,6]. While MVPA entails beneficial health effects, large amounts of time spent sedentarily can be harmful and have been suggested as a potential risk factor for functional disability in older adults [7,8]. Despite some contradictory results [3,9], most studies indicate that reducing the time spent in sedentary activities may be relevant for the prevention of adverse health outcomes [10,11,12], with previous investigations showing positive associations of breaking the sedentary time up and limiting consecutive minutes of sedentary time (prolonged bouts) with BMD [5] and physical function [8,11,12].

Older adults are the most sedentary group in the population, spending approximately 65 to 80% (9 to 10 h/day) of their waking hours engaged in sedentary pursuits [7,13,14] with prolonged bouts of sedentary behavior [15].

Currently, there is a paucity of research assessing the associations of sedentary patterns (breaks and bouts) with BMD and physical function in older adults [5,11], especially in older men [4]. Moreover, longitudinal studies analyzing these associations are scarce [16,17,18]. Thus, the aim of this study was to verify the cross-sectional and prospective associations between objectively measured MVPA and sedentary patterns with BMD and physical function in older adults in a 24-month cohort.

## 2. Methods

### 2.1. Study Design and Participants’ Recruitment

#### 2.1.1. Study Design

The present study is a cohort study performed between January 2015 and February 2017 in the city of Presidente Prudente, São Paulo State, Brazil. The main goal was to analyze the influence of physical activity on sarcopenia, sarcopenic obesity, and functional disability in older adults. In this paper, we chose to present cross-sectional and prospective data.

#### 2.1.2. Study Population

**Figure 1:**
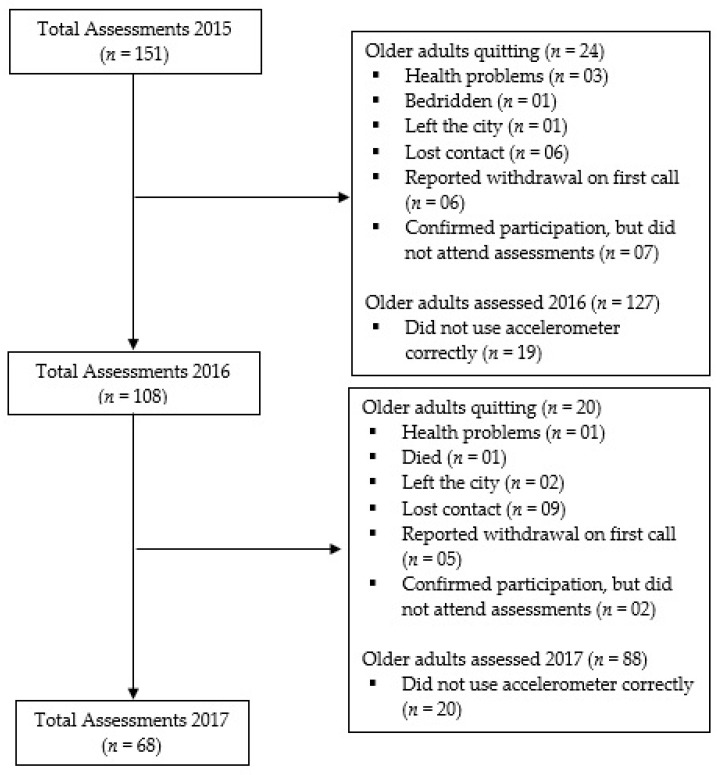
Flowchart of the older adults’ participation during the study.

Sample selection was performed by convenience sampling. The inclusion criterion was as follows: older adults aged 60 years or older living in Presidente Prudente who attended all the evaluations at the laboratory. The exclusion criteria consisted of the following factors: living at a long-term institution; presence of a disease that could lead to muscle mass reduction, such as cancer, HIV/AIDS, tuberculosis, and chronic kidney disease. A minimum sample size of 99 participants was identified based on the expected correlation coefficient of 0.287 between appendicular lean mass and physical activity [19] using the power of 80% and the 5% alpha level. A total of 395 older adults aged 60–85 years were recruited and assessed. For the current study, we only included the participants who had valid accelerometry data. At the baseline, the older adults were randomly selected (one of two persons) to use the accelerometer. This way, 202 participants were selected to use the accelerometer. Of the 202 participants, 15 refused to use the equipment and 36 did not have at least three full valid days of measurement; thus, 151 participants were considered valid and used in the baseline cross-sectional analyses. In the following years (intermediate and final evaluations), phone calls were made to all older adults who participated in the baseline assessments. Of the 151 older adults with valid accelerometry data at the baseline, 68 completed the 24-month follow-up (Figure 1). We posteriorly calculated the power of the test utilized to identify associations between exposures and outcome variables included in this study and found the test power values higher than 76%.

Data collection was performed at the Center for Studies and Laboratory of Assessment and Motor Activity Prescription (CELAPAM), Department of Physical Education, FCT/UNESP. The participants who accepted the invitation to participate in the project signed the informed consent forms. All the protocols were reviewed and approved by the Ethics Research Committee (Process CAEE 26058114.3.0000.5402).

### 2.2. Bone Mineral Density

BMD of the proximal femur and lumbar spine was assessed using dual-energy X-ray absorptiometry (DXA) (Lunar DPX-NT 4.7, General Electric Healthcare, Buckinghamshire, UK). All the examinations were performed according to the manufacturer’s recommendations. The presence of osteopenia or osteoporosis in the proximal femur or lumbar spine (L1-L4) was defined as T-score values ≤ −1.0 or ≤−2.5, respectively [20].

### 2.3. Physical Function

Physical function was estimated using three functional tests: handgrip strength, gait speed, and timed up and go tests as described below.

#### 2.3.1. Handgrip Strength

The handgrip strength was measured using a Camry digital dynamometer model EH101 (Guangdong, China). The test was performed with the participant sitting in a chair without arm support, having their shoulder slightly adducted and elbow of the dominant arm flexed at 90° with the forearm and wrist in the neutral position. The participants were instructed to press the dynamometer as strongly as possible twice at intervals of one minute between each attempt. The highest handgrip strength value (kg) was recorded [21]. Previous test–retest assessments of 16 older men and women measured 24–48 h apart resulted in a standard error of measurement (SEM) of 0.74 kg with the intra-class correlation coefficient (ICC) of 0.97.

#### 2.3.2. Gait Speed

The 4-m walk test was used to assess the participants’ gait speed. The participants were advised to walk in a natural way as if walking indoors. Any habitual apparatus for walking (walking stick, walker, etc.) could be used [22]. The walk test was performed twice and the test with the shortest time (in seconds) was recorded. The SEMs for the 4-m walk test was 0.06 s, with the ICC of 0.95.

#### 2.3.3. Timed Up and Go Test

The timed up and go (TUG) test was performed to evaluate gait speed and dynamic balance (time in seconds). This test consisted of the participant sitting in a stable chair with arm support (hips and back completely leaning on the seat), getting up from the chair, walking three meters, turning around, returning, and sitting back in the chair. The individual could use the arms of the chair to move from the sitting position to the standing position and vice versa [23]. For this test, any apparatus for walking (walking stick, walker, etc.) could be used, and the participant could stop and rest throughout the test. However, they could not be helped by another person or sit down during the course. The SEM for the TUG test was 0.11 s, with the ICC of 0.94.

### 2.4. Physical Activity and Sedentary Patterns

Actigraph GT3X (Actigraph LLC, Pensacola, FL, USA) accelerometer motion sensors were used to estimate daily MVPA and sedentary patterns. Older adults were instructed to wear accelerometers attached to the waist for five consecutive days during all waking hours of the day, except when sleeping and when performing water-based activities (personal hygiene or aquatic activities).

For data analysis, the ActiLife6 software (Actigraph LLC, Pensacola, FL, USA) was used. Only days with at least 600 min were considered valid. Periods of 60 consecutive minutes of zero counts were considered the non-wear time [24]. To be included in the analyses, each participant needed to have at least three full days of monitoring (including one weekend day).

Each minute in which the accelerometer counts were below 100 was considered sedentary time. Total sedentary time was the sum of all sedentary minutes when the accelerometer was worn. A bout was considered a specific period of continuous sedentary time in which the accelerometer count was less than 100 counts/min with no interruption. Bouts of continuous sedentary time (counts/minute < 100) of 20 to 30 min, 30 to 60 min, and >60 min were used in the analyses.

A break in sedentary time was defined as an interruption (lasting at least 1 min) in sedentary time in which the count was above 100 counts/min.

The cut points for defining the intensity of physical activity were analyzed following the recommendation established by Troiano et al. [25]. Low intensity physical activity was defined as values between 100 and 2019 counts/min, moderate physical activity was defined as values between 2020 and 5998 counts/min, and vigorous physical activity was defined as over 5999 counts/min. For this particular study, moderate and vigorous activity was jointly referred to as the MVPA.

### 2.5. Covariates

#### 2.5.1. Anthropometric Measurements

Body mass and stature were measured using an electronic scale (Filizola^®^ Antropométrica, São Paulo, Brazil) and a fixed stadiometer (Sanny^®^, standard model, São Bernardo do Campo, Brazil), respectively. Body mass index (weight (kg)/height^2^ (m)) was calculated.

Information regarding such variables as age, sex, ethnicity, smoking, diseases (hypertension, diabetes, dyslipidemia, and thyroid diseases), and income (monthly salary) of the study population was obtained through a self-reported interview.

#### 2.5.2. Statistical Analysis

The prevalence of osteopenia and osteoporosis in both analyses (cross-sectional and prospective) according to sex was analyzed using the chi-squared test. Descriptive statistics consisted of the mean and standard deviation for all the relevant variables. The means for each variable were compared between sexes using an independent *t*-test. Associations with BMD and physical function were initially explored using the Pearson’s correlation coefficients. Covariates with the *p*-value < 0.05 were included in the linear regression models, which were used to assess the relationships of MVPA and sedentary patterns with BMD and physical function. The results are reported as unstandardized β-coefficients with 95% confidence intervals. Cross-sectional regression analyses were performed for the baseline data controlling for age, BMI, osteopenia and osteoporosis, and MVPA when sedentary patterns (bouts or breaks) were the exposure of interest or the total sedentary time when MVPA was the exposure of interest. For the prospective analyses, the changes for both exposures and outcomes were calculated (follow-up minus baseline) and linear regression models were adjusted for the baseline age, BMI, osteopenia and osteoporosis, and the aimed exposure and outcome, as well as MVPA (changes) when sedentary patterns were the exposure of interest or the total sedentary time (changes) when MVPA was the exposure of interest. Statistical analysis were performed using SPSS software (SPSS Inc., Chicago, IL, USA) version 23.0 and the significance level was set at 5%.

## 3. Results

The participants’ characteristics are shown in Table 1. Significant differences were found between men and women for weight, height, BMI, handgrip strength, femur and spine BMD, femur and spine T-score, breaks in sedentary time (BST), and MVPA.

In cross-sectional analyses, 84.1% of the older men had a normal total proximal femur T-score, 13.6% had osteopenia, and 2.3% had osteoporosis. Among older women, 44.9% had a normal total proximal femur T-score, 49.5% had osteopenia, and 5.6% had osteoporosis.

Regarding the lumbar spine T-score, 75.0%, 20.5%, and 4.5% of the older men had a normal lumbar spine T-score, osteopenia, and osteoporosis, respectively. Among older women, the observed values were 38.3%, 46.7% and 15% for a normal lumbar spine T-score, osteopenia, and osteoporosis, respectively, with older women showing higher prevalence of osteopenia and osteoporosis in both regions (*p* ≤ 0.001).

Cross-sectional analyses of the relationships of MVPA and sedentary patterns with BMD controlling for sex are presented in Table 2. No cross-sectional associations were found at the baseline in either group (male and female).

Cross-sectional analyses of the relationship of MVPA and sedentary patterns with physical function controlling for sex are presented in Table 3. In the female group, negative associations were found for sedentary bouts >60 min with handgrip strength and the TUG test. Furthermore, negative associations were also found for sedentary bouts of 20 to 30 min with the handgrip strength test. No associations were found for the male group.

In prospective analyses, 73.7% of the older men had a normal total proximal femur T-score, 21.0% had osteopenia, and 5.3% had osteoporosis. Among older women, 38.8% had a normal total proximal femur T-score, 55.1% had osteopenia, and 6.1% had osteoporosis.

Regarding the lumbar spine T-score, 78.9%, 21.1%, and 0% of the older men had a normal lumbar spine T-score, osteopenia, and osteoporosis, respectively. In older women, the observed values were 26.5%, 57.2%, and 16.3% for a normal lumbar spine T-score, osteopenia, and osteoporosis, respectively, with older women showing higher prevalence of osteopenia and osteoporosis in both regions (*p* ≤ 0.05).

Table 4 presents the prospective analyses for the associations between the mean changes for MVPA and sedentary patterns with BMD controlling for sex. In the female group, negative associations between the changes in bouts (20 to 30 min and >60 min) and lumbar spine BMD and lumbar spine T-score were found independently of the baseline exposure and outcome, age, BMI, osteopenia or osteoporosis (at the baseline), and MVPA (changes).

Table 5 presents the prospective analyses for the associations between the mean changes for MVPA and sedentary patterns with physical function outcomes controlling for sex. No prospective associations were found between the mean changes for MVPA and sedentary patterns with physical function outcomes.

## 4. Discussion

The present study found that in older women, sedentary patterns were cross-sectionally associated with strength, and with BMD in a prospective manner, regardless of changes in MVPA. In particular, prolonged bouts of sedentary time “>60 min” and “20 to 30 min and >60 min” were inversely associated with handgrip strength when considering the cross-sectional data and with spine BMD in prospective observations, respectively. Interestingly, MVPA was not associated with physical function or bone outcomes in any of the performed analyses. These results suggest that in older women, avoiding prolonged time in sedentary pursuits (20 to 30 min and >60 min) may have beneficial effects on spine BMD.

It is known that prolonged sitting is detrimental to bone health due to the absence of the contractile action of the skeletal muscle on bone as well as the absence of gravitational load on the skeleton [26]. According to the mechanostat theory, bone has a mechanical set point such that below a certain threshold of mechanical strain or load, bone is resorbed [27]. Thus, the relationship between the sedentary behavior and the reduction of bone mass is likely a result of decreased mechanical strain on bone leading to increased bone reabsorption [27]. Previous cross-sectional investigations observed that uninterrupted minutes of sedentary behavior are related to unfavorable total proximal femur BMD in adults and older women regardless of the amount of MVPA they performed [4,5].

Although sedentary time has been negatively associated with bone health, the exact amount of time in which one has to spend in a sedentary behavior for deleterious effects to occur on BMD remains uncertain [4]. In the present study, we found no cross-sectional associations between the time spent in sedentary bouts with bone BMD. We did, however, observe that the change in the amount of time spent in prolonged sedentary bouts (20 to 30 min and >60 min) over a 24-month time period was inversely associated with lumbar spine BMD in older women independent of changes in MVPA levels. Thus, our results point to the importance of encouraging older women who are most affected by bone diseases (osteopenia and osteoporosis) compared to men [28,29] to reduce prolonged time spent in sedentary behavior such that sedentary bouts do not exceed periods longer than 20 min without an interruption, as this may result in beneficial changes in BMD.

On the other hand, there is still no consensus regarding the relationship between BMD and sedentary time in older men. Hind et al. [30] found that total sedentary time was negatively associated with spine BMD and similar results were found previously by Rodríguez–Gomez et al. [31] in regional sites (arm and pelvic). However, in a prospective study, this association was not observed [32] and this result is similar to that found in our study, since there were no cross-sectional or prospective associations between BMD and sedentary time (bouts) in older men. A possible explanation for these discrepancies in results may be due to the characteristics of the samples, since in the studies of Hind et al. [30] and Rodríguez–Gomez et al. [31], older men spent more time sedentarily than older women.

Beyond sedentary bouts, the number of times people break the sedentary time up is an important sedentary pattern to be considered. Braun et al. [5] found a positive association for the number of breaks in sedentary time with BMD of the femoral neck in postmenopausal women in a cross-sectional observation. Contrary to Braun et al. [5], the present study did not observe any cross-sectional or prospective association between the number of breaks in sedentary time and BMD in either men or women. Thus, more longitudinal studies with older adults of both sexes with longer periods of follow-up are needed to confirm such results.

Muscle strength is one of the most important components of physical fitness for performing activities of daily living (ADL) and maintaining a functional capacity in older adults. Reducing the time in activities that stimulate muscle contraction can affect the amount of muscle mass and, consequently, muscle quality [6,17,33]. Furthermore, this mechanical tension is also related to bone quality, as it is a key regulator of osteoblast and osteoclast activity. Bone has an intrinsic ability to adapt its morphology by adding new bone to withstand increased amounts of loading and by removing bone in response to unloading or disuse. How the osteocytes sense the mechanical loads and coordinate adaptive alterations in bone mass and architecture is not yet completely understood, but it is known that mechanical loads placed on bones generate several stimuli that could be detected by osteocytes. These include physical deformation of the bone matrix itself, load-induced flow of the canalicular fluid through the lacuno–canalicular network, and electrical streaming potentials generated from the ionic fluid flowing past the charged surfaces of the lacuno–canalicular channels [34].

In the appendicular skeleton, the trabecular bone transfers mechanical loads from the articular surface to the cortical bone, whereas in vertebral bodies, it represents the main load-bearing structure. Mechanical properties of the bone tissue and architecture of the trabecular bone are two main factors that determine the mechanical properties of the trabecular bone. Fragility fractures that arise in the context of metabolic bone diseases such as osteoporosis usually occur in regions of the trabecular bone [34].

In cross-sectional analyses within this study, negative associations were found for sedentary bouts >60 min with handgrip strength in older women. However, in our prospective analysis, we found no associations between sedentary bouts and muscle strength, which is similar to the result reported by Keevil et al. [18] in a cohort of adults and older adults of both sexes. On the contrary, Hamer and Stamatakis [17] and Reid et al. [6] both reported that the associations between sedentary activities and muscle strength are context-specific (TV watching). However, it should be noted that the contradicting findings may be explained by differences in the dimension of sedentary time that was used (i.e., leisure, total time, bouts of specific lengths), as well as the measurement instrument utilized (i.e., subjective or objective).

Regarding the breaks in sedentary time, we did not observe a significant relationship for the number of breaks in sedentary time with physical function in older adults. These results differ from those previously found by Sardinha et al. [11] and van der Velde [9]. Some possible reasons that may explain these inconsistencies may be related to participant characteristics such as the older adults in the present study being older (mean age: 70 years) than in the study by van der Velde [9] (mean age: 60 years). Another reason can be that our participants had lower physical function and were more sedentary or the specific tests that were used in our study when comparing to the ones used in previous investigations for measuring physical function [9,11].

Some limitations to be mentioned in the present study are the fact that some parameters that may be related to BMD have not been investigated, such as education level, calcium, vitamin D, nutrition, medication, alcohol, and bone fractures. In addition, a reduced sample and the fact that accelerometers are not sensitive to detect all activities, such as biking, standing, and upper body movement, may also be considered relevant limitations. Accelerometers are not able to identify the moment of transition between sitting and standing [35]. Nevertheless, previous studies used this method to assess the associations between sedentary patterns and health outcomes. Even though periods of 60 consecutive minutes of zero counts were considered the non-wear time to avoid an overestimation of sedentary time and the fact that older adults were told to remove the devices when sleeping, one must not disregard that some of them may have kept the accelerometers on while sleeping and that some sleeping time (<60 min) could have been measured as sedentary time. Thus, a slight overestimation of the total sedentary time may have happened.

A major strength of this study is its novelty in the sense that we investigated beyond the simple associations of the total sedentary time with BMD and physical function in older adults and looked into the relationship of sedentary patterns (breaks and bouts) with these outcomes both from a cross-sectional perspective and considering a 24-month longitudinal perspective. In addition, despite the aforementioned limitations in the use of accelerometry to assess sedentary patterns and MVPA, it is a widely used objective method within the physical activity research field, thus allowing comparison with previous studies.

## 5. Conclusions

In older women, sedentary patterns are cross-sectionally associated with handgrip strength and prospectively associated with BMD independent of MVPA. The findings from the present study indicate that, in the long term, limiting continuous prolonged sedentary bouts, especially longer than 60 min, may have beneficial effects on BMD. Thus, in addition to some preventive strategies such as engagement in physical activity, intervention programs must aim to reduce sedentary time in the daily routine of this specific population, as it can promote benefits to BMD.

## Figures and Tables

**Table 1:** Mean and standard deviation values for the participants’ characteristics at the baseline according to sex.

Variables	Total (*n* = 151)	Male (*n* = 44)	Female (*n* = 107)
Age (years)	69.4 ± 6.5	70.5 ± 6.9	69.0 ± 6.3
Weight (kg) **	71.5 ± 14.7	78.9 ± 13.4	68.4 ± 14.1
Height (cm) **	159.1 ± 8.4	167.6 ± 5.5	155.6 ± 6.7
BMI (kg/m^2^)	28.2 ± 5.1	28.1 ± 4.4	28.2 ± 5.4
Handgrip (kg) **	26.2 ± 8.2	35.7 ± 6.8	22.4 ± 4.9
Gait speed (m/s)	1.0 ± 0.2	1.1 ± 0.2	1.0 ± 0.2
TUG (s)	9.6 ± 2.4	9.4 ± 2.1	9.6 ± 2.6
Femur BMD (g/cm^2^) **	0.9 ± 0.2	1.1 ± 0.2	0.9 ± 0.1
Femur T-score **	−0.7 ± 1.1	−0.1 ± 1.2	−1.0 ± 1.0
Spine BMD (g/cm^2^) **	1.1 ± 0.2	1.3 ± 0.2	1.1 ± 0.2
Spine T-score **	−0.8 ± 1.2	0.4 ± 1.9	−1.3 ± 1.4
Sedentary time (min/day)	543.8 ± 128.6	535.9 ± 110.9	547.1 ± 135.5
BST (number/day) *	94.5 ± 15.6	88.5 ± 15.6	97.0 ± 15.0
MVPA (min/day) *	21.1 ± 22.5	29.7 ± 28.7	17.5 ± 18.4
Continuous sedentary bouts (number/day)			
20 to 30 min	7.1 ± 3.8	7.5 ± 3.4	6.9 ± 3.9
30 to 60 min	3.1 ± 2.0	3.4 ± 1.7	3.0 ± 2.1
>60 min	0.6 ± 0.6	0.6 ± 0.6	0.6 ± 0.6

Note: BMI = body mass index; TUG = timed up and go test; BMD = bone mineral density; BST = breaks in sedentary time; MVPA = moderate and vigorous physical activity; * significant differences between groups, *p* < 0.05; ** significant differences between groups, *p* ≤ 0.001.

**Table 2:** Cross-sectional associations of the MVPA and sedentary patterns with BMD (proximal total femur and lumbar spine) controlling for sex.

Variables	Male (*n* = 44)		Female (*n* = 107)	
	β (95% CI) ^a^	β (95% CI) ^b^	β (95% CI) ^a^	β (95% CI) ^b^
**Femur BMD (g/cm^2^)**				
Bouts (20 to 30 min)	0.00 (−0.01, 0.02)	0.00 (−0.01, 0.00)	−0.00 (−0.01, 0.00)	−0.00 (−0.01, 0.0)
Bouts (30 to 60 min)	0.01 (−0.02, 0.04)	0.00 (−0.02, 0.03)	−0.01 (−0.02, 0.00)	−0.01 (−0.02, 0.00)
Bouts (>60 min)	−0.02 (−0.11, 0.07)	−0.04 (−0.11, 0.04)	−0.02 (−0.06, 0.00)	−0.02 (−0.05, 0.01)
BST (number/day)	0.00 (−0.00, 0.00)	0.00 (−0.00, 0.00)	0.00 (−0.00, 0.00)	0.00 (−0.00, 0.00)
MVPA (min/day)	0.00 (−0.00, 0.00)	0.00 (−0.00, 0.00)	0.00 (−0.00, 0.00)	0.00 (−0.00, 0.00)
**Femur T-score**				
Bouts (20 to 30 min)	0.03 (−0.07, 0.14)	0.00 (−0.09, 0.10)	−0.03 (−0.07, 0.01)	−0.03 (−0.07, 0.01)
Bouts (30 to 60 min)	0.06 (−0.14, 0.26)	0.03 (−0.15, 0.21)	−0.05 (−0.12, 0.03)	−0.05 (−0.13, 0.02)
Bouts (>60 min)	−0.13 (−0.73, 0.47)	−0.25 (−0.78, 0.27)	−0.19 (−0.43, 0.05)	−0.12 (−0.36, 0.12)
BST (number/day)	0.00 (−0.02, 0.03)	−0.00 (−0.02, 0.02)	0.01 (−0.00, 0.02)	0.01 (−0.00, 0.02)
MVPA (min/day)	0.00 (−0.01, 0.01)	0.00 (−0.01, 0.01)	0.00 (−0.01, 0.01)	0.00 (−0.00, 0.01)
**Spine BMD (g/cm^2^)**				
Bouts (20 to 30 min)	0.02 (−0.00, 0.04)	0.01 (−0.00, 0.03)	0.00 (−0.01, 0.01)	0.00 (−0.00, 0.01)
Bouts (30 to 60 min)	0.02 (−0.02, 0.06)	0.01 (−0.02, 0.04)	0.00 (−0.01, 0.02)	0.01 (−0.01, 0.01)
Bouts (>60 min)	0.00 (−0.12, 0.12)	−0.02 (−0.12, 0.07)	−0.01 (−0.06, 0.04)	0.00 (−0.03, 0.04)
BST (number/day)	−0.00 (−0.01, 0.00)	−0.00 (−0.01, 0.00)	0.00 (−0.00, 0.00)	0.00 (−0.00, 0.00)
MVPA (min/day)	0.00 (−0.00, 0.00)	0.00 (−0.00, 0.00)	0.00 (−0.00, 0.00)	0.00 (−0.00, 0.00)
**Spine T-score**				
Bouts (20 to 30 min)	0.14 (−0.04, 0.32)	0.10 (−0.03, 0.24)	0.01 (−0.05, 0.08)	0.01 (−0.04, 0.06)
Bouts (30 to 60 min)	0.14 (−0.19, 0.48)	0.14 (−0.16, 0.36)	0.01 (−0.12, 0.13)	0.00 (−0.09, 0.09)
Bouts (>60 min)	0.04 (−0.96, 1.03)	−0.20 (−0.98, 0.58)	−0.08 (−0.48, 0.32)	0.05 (−0.25, 0.34)
BST (number/day)	−0.01 (−0.05, 0.03)	−0.02 (−0.05, 0.01)	0.00 (−0.02, 0.02)	0.01 (−0.01, 0.01)
MVPA (min/day)	0.01 (−0.01, 0.04)	0.00 (−0.02, 0.02)	0.00 (−0.02, 0.02)	−0.00 (−0.01, 0.01)

Note: BST = breaks in sedentary time; BMD = bone mineral density; MVPA = moderate and vigorous physical activity; TUG = timed up and go test; β = unstandardized coefficients. ^a^ The models were adjusted for age, BMI, and MVPA or the total sedentary time (depending on the aimed exposure). ^b^ The models were adjusted for osteopenia or osteoporosis and MVPA or the total sedentary time (depending on the aimed exposure).

**Table 3:** Cross-sectional associations of MVPA and sedentary patterns with physical function controlling for sex.

Functional Tests	Male (*n* = 44)		Female (*n* = 107)	
	β (95% CI) ^a^	β (95% CI) ^b^	β (95% CI) ^a^	β (95% CI) ^b^
**Handgrip (kg)**				
Bouts (20 to 30 min)	0.07 (−0.49, 0.63)	0.09 (−0.47, 0.66)	−0.25 (−0.48, −0.01) *	−0.24 (−0.48, 0.00)
Bouts (30 to 60 min)	0.19 (−0.86, 1.23)	0.24 (−0.82, 1.30)	−0.42 (−0.85, 0.01)	−0.41 (−0.85, 0.04)
Bouts (>60 min)	0.73 (−2.34, 3.80)	0.79 (−2.30, 3.89)	−2.05 (−3.41, −0.68) *	−2.03 (−3.43, −0.63) *
BST (number/day)	0.01 (−0.11, 0.13)	0.02 (−0.10, 0.15)	0.02 (−0.04, 0.08)	0.02 (−0.04, 0.09)
MVPA (min/day)	0.02 (−0.04, 0.09)	0.03 (−0.04, 0.10)	0.02 (−0.04, 0.07)	0.01 (−0.05, 0.06)
**Gait speed (m/s)**				
Bouts (20 to 30 min)	0.00 (−0.01, 0.02)	0.00 (−0.02, 0.02)	−0.00 (−0.01, 0.01)	−0.00 (−0.01, 0.01)
Bouts (30 to 60 min)	0.00 (−0.03, 0.04)	−0.00 (−0.04, 0.03)	−0.00 (−0.02, 0.02)	−0.00 (−0.02, 0.02)
Bouts (>60 min)	0.02 (−0.08, 0.12)	0.02 (−0.08, 0.12)	−0.03 (−0.09, 0.04)	−0.03 (−0.10, 0.04)
BST (number/day)	0.00 (−0.00, 0.00)	0.00 (−0.00, 0.00)	0.00 (−0.00, 0.00)	0.00 (−0.00, 0.00)
MVPA (min/day)	−0.00 (−0.00, 0.00)	−0.00 (−0.00, 0.00)	−0.00 (−0.00, 0.00)	0.00 (−0.00, 0.00)
**TUG (s)**				
Bouts (20 to 30 min)	0.05 (−0.14, 0.24)	0.06 (−0.13, 0.26)	0.11 (−0.00, 0.23)	0.10 (−0.02, 0.22)
Bouts (30 to 60 min)	0.07 (−0.29, 0.43)	0.10 (−0.26, 0.46)	0.19 (−0.02, 0.40)	0.17 (−0.05, 0.38)
Bouts (>60 min)	0.78 (−0.25, 1.82)	0.82 (−0.20, 1.85)	0.75 (0.07, 1.43) *	0.69 (−0.01, 1.39)
BST (number/day)	−0.02 (−0.07, 0.02)	−0.02 (−0.06, 0.02)	−0.01 (−0.04, 0.02)	−0.02 (−0.05, 0.01)
MVPA (min/day)	0.01 (−0.01, 0.04)	0.02 (−0.01, 0.04)	−0.02 (−0.04, 0.01)	−0.01 (−0.04, 0.02)

Note: BST = breaks in sedentary time; MVPA = moderate and vigorous physical activity; TUG = timed up and go test; β = unstandardized coefficients; * *p* < 0.05. ^a^ The models were adjusted for age and BMI. ^b^ The models were adjusted for age and BMI plus MVPA or the total sedentary time (depending on the aimed exposure).

**Table 4:** Prospective associations of MVPA and sedentary patterns with BMD (mean changes) controlling for sex.

Variables	Male (*n* = 19)		Female (*n* = 49)	
	β (95% CI) ^a^	β (95% CI) ^b^	β (95% CI) ^a^	β (95% CI) ^b^
**Femur BMD (g/cm^2^)**				
Bouts (20 to 30 min)	0.00 (−0.02, 0.02)	−0.00 (−0.02, 0.01)	−0.00 (−0.00, 0.00)	0.00 (−0.00, 0.00)
Bouts (30 to 60 min)	0.01 (−0.04, 0.05)	0.01 (−0.03, 0.03)	0.00 (−0.00, 0.00)	0.00 (−0.00, 0.00)
Bouts (>60 min)	−0.02 (−0.11, 0.08)	−0.02 (−0.10, 0.05)	−0.00 (−0.01, 0.01)	−0.00 (−0.01, 0.01)
BST (number/day)	0.00 (−0.00, 0.01)	0.00 (−0.00, 0.00)	0.00 (−0.00, 0.00)	0.00 (−0.00, 0.00)
MVPA (min/day)	0.00 (−0.01, 0.00)	0.00 (−0.01, 0.00)	0.00 (−0.00, 0.00)	0.00 (−0.00, 0.00)
**Femur T-score**				
Bouts (20 to 30 min)	0.02 (−0.11, 0.16)	−0.00 (−0.10, 0.10)	−0.00 (−0.02, 0.01)	−0.00 (−0.02, 0.01)
Bouts (30 to 60 min)	0.05 (−0.23, 0.34)	0.01 (−0.20, 0.22)	0.00 (−0.03, 0.03)	0.00 (−0.03, 0.03)
Bouts (>60 min)	−0.03 (−0.66, 0.61)	−0.08 (−0.57, 0.40)	−0.02 (−0.11, 0.06)	−0.03 (−0.11, 0.05)
BST (number/day)	0.02 (−0.01, 0.05)	0.01 (−0.01, 0.03)	0.00 (−0.00, 0.00)	0.00 (−0.00, 0.00)
MVPA (min/day)	−0.01 (−0.04, 0.03)	−0.01 (−0.04, 0.02)	0.00 (−0.00, 0.01)	0.00 (−0.00, 0.01)
**Spine BMD (g/cm^2^)**				
Bouts (20 to 30 min)	−0.00 (−0.01, 0.01)	−0.00 (−0.01, 0.01)	−0.01 (−0.01, −0.00) *	−0.01 (−0.01, −0.00) *
Bouts (30 to 60 min)	−0.01 (−0.03, 0.02)	−0.01 (−0.02, 0.01)	−0.01 (−0.02, 0.00)	−0.01 (−0.02, 0.00)
Bouts (>60 min)	−0.01 (−0.06, 0.04)	−0.01 (−0.05, 0.03)	−0.04 (−0.07, −0.01) *	−0.03 (−0.06, −0.01) *
BST (number/day)	0.00 (−0.00, 0.00)	0.00 (−0.00, 0.00)	0.00 (−0.00, 0.00)	0.00 (−0.00, 0.00)
MVPA (min/day)	0.00 (−0.00, 0.00)	0.00 (−0.00, 0.00)	−0.00 (−0.00, 0.00)	−0.00 (−0.00, 0.00)
**Spine T-score**				
Bouts (20 to 30 min)	−0.00 (−0.01, 0.01)	−0.02 (−0.09, 0.04)	−0.01 (−0.01, −0.00) *	−0.06 (−0.10, −0.01) *
Bouts (30 to 60 min)	−0.03 (−0.21, 0.16)	−0.04 (−0.19, 0.11)	−0.09 (−0.19, 0.01)	−0.09 (−0.18, 0.00)
Bouts (>60 min)	−0.03 (−0.46, 0.40)	−0.06 (−0.40, 0.38)	−0.31 (−0.55, −0.07) *	−0.27 (−0.49, −0.04) *
BST (number/day)	0.00 (−0.01, 0.02)	0.00 (−0.01, 0.02)	0.00 (−0.01, 0.01)	−0.00 (−0.01, 0.01)
MVPA (min/day)	−0.01 (−0.02, 0.02)	−0.00 (−0.02, 0.02)	−0.01 (−0.02, 0.00)	−0.01 (−0.02, 0.00)

Note: BST = breaks in sedentary time; BMD = bone mineral density; MVPA = moderate and vigorous physical activity; TUG = timed up and go test; β = unstandardized coefficients; * *p* < 0.05. ^a^ The models were adjusted for the baseline exposure and outcome, age, BMI, and MVPA or the total sedentary time (changes) (depending on the aimed exposure). ^b^ The models were adjusted for the baseline exposure and outcome, osteopenia or osteoporosis, and MVPA or total sedentary time (changes) (depending on the aimed exposure).

**Table 5:** Prospective associations of MVPA and sedentary patterns with physical function (mean changes) controlling for sex.

Functional Tests	Male (*n* = 19)		Female (*n* = 49)	
	β (95% CI) ^a^	β (95% CI) ^b^	β (95% CI) ^a^	β (95% CI) ^b^
**Handgrip (kg)**				
Bouts (20 to 30 min)	0.04 (−0.45, 0.52)	0.03 (−0.48, 0.54)	0.06 (−0.23, 0.34)	0.06 (−0.23, 0.36)
Bouts (30 to 60 min)	−0.27 (−1.64, 1.09)	−0.33 (−1.75, 1.09)	−0.10 (−0.66, 0.47)	−0.10 (−0.69, 0.49)
Bouts (>60 min)	0.02 (−3.48, 3.53)	−0.02 (−3.57, 3.52)	0.03 (−1.52, 1.58)	0.04 (−1.58, 1.66)
BST (number/day)	0.07 (−0.03, 0.17)	0.06 (−0.04, 0.17)	0.04 (−0.02, 0.10)	0.04 (−0.02, 0.10)
MVPA (min/day)	−0.08 (−0.21, 0.04)	−0.08 (−0.20, 0.04)	−0.05 (−0.14, 0.03)	−0.05 (−0.14, 0.03)
**Gait speed (m/s)**				
Bouts (20 to 30 min)	−0.01 (−0.04, 0.03)	−0.00 (−0.04, 0.03)	−0.01 (−0.02, 0.01)	−0.01 (−0.02, 0.01)
Bouts (30 to 60 min)	−0.02 (−0.10, 0.06)	−0.02 (−0.10, 0.06)	−0.01 (−0.04, 0.01)	−0.01 (−0.04, 0.01)
Bouts (>60 min)	−0.03 (−0.22, 0.15)	−0.03 (−0.22, 0.15)	0.01 (−0.07, 0.09)	0.01 (−0.07, 0.09)
BST (number/day)	0.00 (−0.01, 0.01)	−0.00 (−0.01, 0.01)	0.00 (−0.00, 0.00)	0.00 (−0.00, 0.00)
MVPA (min/day)	0.01 (−0.00, 0.02)	0.01 (−0.00, 0.02)	0.00 (−0.00, 0.00)	0.00 (−0.00, 0.00)
**TUG (s)**				
Bouts (20 to 30 min)	−0.04 (−0.47, 0.40)	−0.04 (−0.50, 0.41)	−0.04 (−0.40, 0.33)	−0.03 (−0.40, 0.35)
Bouts (30 to 60 min)	0.16 (−0.82, 1.14)	0.17 (−0.87, 1.20)	−0.03 (−0.76, 0.70)	0.00 (−0.76, 0.76)
Bouts (>60 min)	−0.11 (−2.25, 2.03)	−0.11 (−2.35, 2.13)	−0.33 (−2.28, 1.62)	−0.29 (−2.31, 1.73)
BST (number/day)	0.00 (−0.11, 0.11)	0.00 (−0.11, 0.12)	−0.02 (−0.09, 0.05)	−0.02 (−0.09, 0.05)
MVPA (min/day)	−0.02 (−0.14, 0.09)	−0.02 (−0.15, 0.10)	0.02 (−0.09, 0.14)	0.02 (−0.11, 0.14)

Note: BST = breaks in sedentary time; MVPA = moderate and vigorous physical activity; TUG = timed up and go test; β = unstandardized coefficients. ^a^ The models were adjusted for the baseline exposure and outcome, age, and BMI. ^b^ The models were adjusted for the same covariates as in model A plus MVPA or the total sedentary time (changes) (depending on the aimed exposure).
